# Bioactive Ingredient Profiling of *Dendrobium officinale*: Plant-Part-Specific Distribution of Key Metabolites and Their Multi-Disease Therapeutic Potential

**DOI:** 10.3390/metabo16010010

**Published:** 2025-12-22

**Authors:** Yue Yang, Yongxin Guan, Shasha Li, Yingchao Xu

**Affiliations:** 1 Department of Chemical and Pharmaceutical Engineering, Chongqing Industry Polytechnic University, Chongqing 401120, China; 2 Guangdong Key Laboratory for New Technology Research of Vegetables, Vegetable Research Institute, Guangdong Academy of Agricultural Sciences, Guangzhou 510640, China

**Keywords:** *Dendrobium officinale*, comparative metabolomics, UHPLC-MS/MS, TCMSP, plant-part-specific differences

## Abstract

**Background/Objectives:** *Dendrobium officinale* is a valuable medicinal orchid. However, the metabolic profiles of its leaves and flowers remain poorly characterized. This highlights the need for comprehensive analysis of stems, leaves, and flowers to reveal plant-part-specific bioactive compounds and expand whole-plant utilization. **Methods:** An integrative metabolomic approach based on UHPLC–MS/MS was employed to systematically characterize secondary metabolite profiles in different parts of *D. officinale*, including stems (DOS), leaves (DOL), and flowers (DOF). **Results:** A total of 761 metabolites, predominantly flavonoids (30.6%), alkaloids (20.2%), phenolic acids (12.2%), and terpenoids (9.3%), were identified. The most abundant metabolites were detected in DOF (634), followed by DOL (598) and DOS (586). Total flavonoid and alkaloid contents were the highest in DOF, reaching 0.86 and 0.62 mg·g^−1^ DW, respectively. Screening identified 74 key active ingredients (KAI) and 83 active pharmaceutical ingredients (API) and demonstrated potential efficacy against six major human diseases. Among these, gardenoside and phloroglucinol were uniquely present in leaves, whereas 12 KAIs and 16 APIs were specific to DOF. Quercetin, a compound associated with more than 90 disease-related entries, was exclusively detected in DOF. Multivariate analyses revealed clear separation among the three plant parts. Furthermore, 15 metabolites with VIP > 1, including pinobanksin and naringenin, exhibited distinct plant-part-specific accumulation patterns. Additionally, potential plant-part-specific biomarkers were identified. **Conclusions:** This study presents a comprehensive plant-part-specific metabolomic profile of *D. officinale*, revealing that its flowers and leaves are particularly enriched in bioactive flavonoids and alkaloids. The findings reveal the remarkable metabolic diversity and functional potential of *D. officinale*, providing essential chemical insights that support the whole plant’s broader medicinal and biotechnological applications.

## 1. Introduction

*Dendrobium officinale* Kimura et Migo, a representative species belonging to Orchidaceae, is highly valued in traditional Chinese medicine (TCM) for its remarkable therapeutic efficacy and pharmacological diversity [1,2]. Traditionally, its stem serves as the principal medicinal plant part. Modern pharmacological studies have demonstrated that the stems possess antioxidant, immunoregulatory, anti-inflammatory, and antitumor effects [3,4,5,6], establishing their well-documented medicinal profile. This broad spectrum of bioactivities has led to its extensive application in TCM and the development of functional health products [7,8]. Despite its widespread application, the commercial exploitation of *D. officinale* focuses on its stems. In practice, other potentially valuable plant parts, such as the leaves and flowers, are typically discarded or remain markedly underutilized, leading to substantial biomass waste and hindering the comprehensive utilization of the plant’s potential [9]. Leaves account for nearly half of the total biomass of *D. officinale* and serve as a significant source of bioactive metabolites with potential applications [10]. However, systematic evaluation of leaves and flowers has been limited, highlighting the need for comprehensive investigations to explore their chemical richness and health-promoting potential.

The concept of comprehensive utilization of medicinal plants has gained increasing attention in recent years, emphasizing the development of multiple plant parts to improve resource efficiency and economic value [11]. The leaves and flowers of *D. officinale* have been reported to accumulate notable levels of flavonoids and alkaloids, which are two classes of compounds widely associated with the plant’s known pharmacological activities [12,13,14]. *D. officinale* leaves exhibit marked pharmacological potential, including hepatoprotective, antioxidant, and anti-inflammatory effects [8,15]. In contrast, flowers exhibit distinct neuroprotective, antihypertensive, and antidepressant effects [16,17]. These findings indicate that *D. officinale* flowers and leaves, like the stems, possess health-promoting properties, supporting their potential for broader pharmaceutical and nutraceutical applications. Furthermore, both leaves and flowers have attracted increasing interest for their potential in the development of functional foods, with applications including teas and other health-oriented products [18,19]. Nevertheless, comprehensive metabolite profiling of these plant parts remains limited, and their phytochemical profiles have yet to be systematically compared.

The advent of widely targeted metabolomics using ultra-high-performance liquid chromatography–tandem mass spectrometry (UHPLC–MS/MS) enables high-throughput, sensitive, and comprehensive profiling of complex plant metabolites, integrating broad coverage with quantitative precision [20,21,22,23,24]. In this study, we applied this approach to systematically analyze the stems, leaves, and flowers of *D. officinale*, revealing plant-part-specific accumulation patterns of key bioactive metabolites. By uncovering the untapped chemical richness of the floral and foliar parts, we provide a scientific foundation for the expanded medicinal and industrial utilization of the entire plant. Consequently, a critical knowledge gap in the phytochemistry of *D. officinale* is filled by this study, and novel avenues for developing pharmaceuticals and value-added products from previously underexploited plant parts are reported.

## 2. Materials and Methods

### 2.1. Plant Materials and Sample Collection

**Figure 1:**
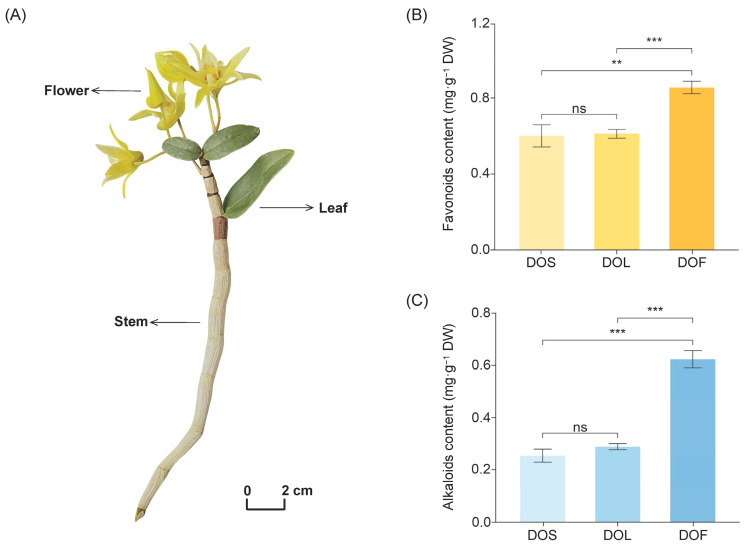
Phenotype and major secondary metabolites in different plant parts of *D. officinale*. (**A**) Morphology of stems (DOS), leaves (DOL), and flowers (DOF). (**B**) Total flavonoid contents in DOS, DOL, and DOF. (**C**) Total alkaloid contents in DOS, DOL, and DOF. Data are expressed as mean ± SD (*n* = 3). Asterisks indicate statistical differences between plant parts using Student’s *t* tests (ns > 0.05, ** *p* < 0.01, *** *p* < 0.001).

*D. officinale* was artificially cultivated at Qiaodongmei Industrial Co., Ltd., Dianjiang County, Chongqing Municipality, China (30°22′ N, 107°23′ E). Plants were maintained under a tree-epiphytic cultivation system for 2 years. The plant material was authenticated as *Dendrobium officinale* by Dr. Shasha Li at Chongqing Industry Polytechnic University based on its characteristic morphological features. The morphology of stems, leaves, and flowers of *D. officinale* is shown in Figure 1A.

Samples of stems, leaves, and flowers (abbreviated as DOS, DOL, and DOF) were collected on 2 June 2024, when Plants were at a mature growth stage with fully developed stems and leaves, and flowers at full bloom. For each plant part, material was harvested from five independent plants, combined to form a homogeneous composite sample, and then divided into three portions to generate three biological replicates. The collected samples were immediately frozen in liquid nitrogen to preserve their integrity and then stored at –80 °C until analysis.

### 2.2. Determination of Total Flavonoid and Total Alkaloid Contents

The total flavonoid content was measured following the procedure reported by Liu et al. with slight modifications [25]. The different plant parts of *D. officinale* were dried and finely ground into powder. Powdered samples (0.02 g) were extracted with 1 mL of 80% methanol by sonication at 60 °C for 30 min, followed by centrifugation at 12,000× *g* for 10 min at 25 °C. The supernatant was adjusted to 1 mL with 80% methanol. For flavonoid determination, 60 μL of the extract was mixed with 25 μL of 5% (*w*/*w*) NaNO_2_, incubated for 6 min, and then treated with 25 μL of 10% (*w*/*w*) AlCl_3_. After another 6 min, 200 μL of 1M NaOH and 190 μL of 80% methanol were added to achieve a final volume of 500 μL. The mixture was incubated at 30 °C for 30 min, and the absorbance was recorded at 510 nm. Quantification was performed using rutin as the reference standard. Calibration standards ranged from 0.125 to 2.5 mg·mL^−1^, and the resulting calibration curve was y = 0.6561x−0.021 (R^2^ = 0.9979).

The total alkaloid content was determined following the procedure of Xie et al. with minor adjustments [26]. About 0.02 g of the powdered sample was extracted in 1 mL of 80% ethanol ultrasonically for 60 min. The extracts were subsequently centrifuged at 8000× *g* and 25 °C for 10 min, and 0.4 mL of the resulting supernatant was evaporated to dryness at 50 °C overnight. The residue was reconstituted in 0.2 mL of potassium hydrogen phthalate buffer (pH 4.5) and sonicated for 5 min. An aliquot (150 µL) was combined with 750 μL of the same buffer and 300 μL of 0.04% bromocresol green solution, and incubated at room temperature for 5 min. Next, 1.5 mL of chloroform was added, followed by vigorous shaking and 40 min incubation at ambient temperature. Finally, 1 mL of the chloroform phase was collected, and absorbance was measured at 416 nm.

### 2.3. Metabolites Analysis Based on Widely Targeted Metabolomic Profiling

Samples were prepared and extracted according to the protocols provided by Metware Biotechnology Co., Ltd. (Wuhan, China) as previously described [27]. The collected samples were freeze-dried using a vacuum lyophilizer (Scientz-100F, Ningbo Scientz Biotechnology Co., Ltd., Ningbo, China) and ground into powder using a grinder (MM 400, Retsch GmbH, Haan, Germany) with zirconia beads at 30 Hz for 1.5 min. Further, 50 mg of the powdered sample was extracted with 1.2 mL of pre-chilled (−20 °C) 70% methanol, agitated by vortexing for 30 s every 30 min for a total of six times, and centrifuged at 12,000× *g* for 3 min. The internal standard 2-chloro-L-phenylalanine was added prior to extraction to correct for variability during sample preparation and analysis.

The supernatant was filtered and immediately subjected to UHPLC–MS/MS analysis. Separation was achieved on a C18 column with a binary mobile phase comprising water (0.1% formic acid, A) and acetonitrile (0.1% formic acid, B). A linear gradient from 5% to 95% B was applied for 9 min and then at 95% B for 1 min. The column temperature was maintained at 40 °C, with a flow rate of 0.3 mL/min and an injection volume of 2 μL. Detection was performed in multiple reaction monitoring (MRM) mode on a quadrupole ion trap tandem mass spectrometer (QTRAP) system.

Metabolites were identified and characterized using MS/MS spectral data, with isotopic peaks and redundant signals removed. Quantitative analysis was carried out using a triple quadrupole (QQQ) mass spectrometer. Peak areas were integrated across all samples, and signals corresponding to the same metabolite were normalized. Metabolic data were processed and analyzed using Analyst 1.6.3 software.

### 2.4. Identification of the Key Active Ingredients and Active Pharmaceutical Ingredients in Dendrobium officinale

Metabolites identified by UHPLC–MS/MS were screened in the Traditional Chinese Medicine Systems Pharmacology Database and Analysis Platform (TCMSP) to identify the key active ingredients (KAI) in *D. officinale* [28]. Screening was based on oral bioavailability (OB) ≥ 5% and drug-likeness (DL) ≥ 0.14 [29]. Furthermore, all identified metabolites were screened to identify active pharmaceutical ingredients (API) associated with six major diseases. The anticancer compounds were retrieved using the CancerHSP database within TCMSP. For the other diseases, including diabetes, Alzheimer’s disease, hypertension, cardiovascular disease, and asthma, the corresponding disease names were entered into the TCMSP database to identify antidiabetic, antihypertensive, anti-cardiovascular, anti-Alzheimer, and anti-asthma ingredients, respectively.

### 2.5. Data Analysis

All compound content data are presented as the mean ± standard deviation (SD) of three independent biological replicates. GraphPad Prism software (version 7.0) was used for statistical analyses. Statistical differences among different plant parts were determined by Student’s *t*-tests, with asterisks indicating the level of significance: * *p* < 0.05, ** *p* < 0.01, *** *p* < 0.001; not significant (ns), *p* > 0.05.

Multivariate statistical analyses, including principal component analysis (PCA), orthogonal partial least squares discriminant analysis (OPLS-DA), hierarchical clustering analysis (HCA), and Pearson correlation analysis (PCC), were performed using R software (Version 4.3.1, R Foundation for Statistical Computing, Vienna, Austria). Partial least squares discriminant analysis (PLS-DA) was conducted with MetaboAnalyst 6.0. Differential metabolites were identified based on VIP ≥ 1 and absolute log2FC ≥ 1 [30]. Metabolite annotation was performed by querying the KEGG Compound database. The identified metabolites were subsequently mapped to the KEGG pathway database for metabolic pathway enrichment analysis [31,32].

## 3. Results

### 3.1. Contents of Total Flavonoids and Alkaloids

Flavonoids and alkaloids are recognized as the primary bioactive constituents in *D. officinale*. Our experimental results further demonstrate a marked enrichment of both flavonoids and alkaloids in DOF (*p* < 0.05), whereas their levels were comparable between DOS and DOL (*p* > 0.05) (Figure 1B,C). The total flavonoid content was approximately 1.4-fold higher in DOF (0.86 mg·g^−1^ DW) than in DOS (0.60 mg·g^−1^ DW) and DOL (0.61 mg·g^−1^ DW). Furthermore, the total alkaloid content in flowers (0.62 mg·g^−1^ DW) was more than twice that in DOS (0.25 mg·g^−1^ DW) and DOL (0.29 mg·g^−1^ DW). These results indicated that both flowers and leaves of *D. officinale* were rich in flavonoids and alkaloids. Flowers exhibited particularly high levels, indicating their potential as valuable sources of bioactive compounds beyond the traditionally used stems.

### 3.2. Detection of Metabolites in Dendrobium officinale

**Figure 2:**
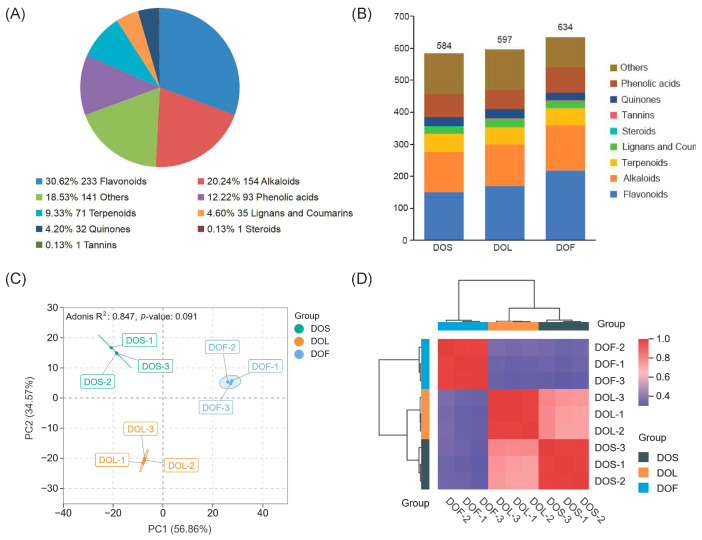
Metabolite profiling and identification in *D. officinale*. (**A**) Classification and relative percentages of all metabolites identified in *D. officinale*. (**B**) Distribution of metabolites in stems, leaves, and flowers. (**C**) PCA score plot of metabolites. (**D**) Correlation heatmaps of metabolites, where colors indicate the strength of Pearson correlation coefficients, with purple representing lower correlations and red representing higher correlations. Sample labels DOS (1–3), DOL (1–3), and DOF (1–3) represent three biological replicates of stems (DOS), leaves (DOL), and flowers (DOF), respectively. Metabolite data were normalized and log-transformed prior to multivariate analysis (*n* = 3).

The secondary metabolites of stems, leaves, and flowers in *D. officinale* were systematically characterized in this study using UHPLC–MS/MS. A mixture of all sample extracts served as quality control (QC) and was analyzed under the same conditions as the test samples. The reliability and reproducibility of the data were verified by QC total ion chromatogram (TIC) plot and sample multi-peak detection (Figure S1). A total of 761 metabolites, including 233 flavonoids, 154 alkaloids, 141 others, 93 phenolic acids, 71 terpenoids, 35 lignans and coumarins, 32 quinones, 1 steroid, and 1 tannin, were identified (Figure 2A). All identified metabolites are summarized in Supplementary Table S1. The distribution of these metabolites among different plant parts of *D. officinale* is illustrated in Figure 2B. DOF contained the highest number of identified metabolites (634), followed by DOL (598) and DOS (586). A Venn diagram revealed 442 metabolites as common to all plant parts (Figure S2A).

PCA was performed to provide an overview of metabolomic profiles. The first two principal components (PC1 and PC2) accounted for 56.86% and 34.57% of the total variance, respectively, together explaining 91.43% of the total variation (Figure 2C). The samples were clearly separated into three distinct groups, with minimal variation observed within each group. The correlation analysis was conducted to assess the similarity of metabolite profiles across all samples. As shown in Figure 2D, metabolite profiles within the stem, leaf, and flower groups were highly correlated, indicating strong intra-group consistency and reliable data reproducibility. In contrast, lower correlation coefficients between different plant parts revealed distinct metabolic differentiation among the stem, leaf, and flower of *D. officinale*. Furthermore, HCA was conducted to investigate metabolic variation patterns among the samples. The results revealed distinct clustering according to different plant parts, with the stems, leaves, and flowers forming clearly separated groups (Figure S2B). This clustering pattern was aligned with the PCA and correlation analyses, confirming substantial plant-part-specific metabolic differentiation in *D. officinale*. These findings also demonstrated consistent clustering and acceptable data stability, supporting further analyses.

### 3.3. Identification of the Key Active Ingredients in Dendrobium officinale

TCMSP was employed to screen for bioactive metabolites in *D. officinale* with potential health-promoting effects. Among the 761 identified metabolites, 171 were the chemical constituents of TCM, as recorded in the TCMSP database. Metabolites were screened using criteria of OB ≥ 5% and DL ≥ 0.14 to further identify KAIs. Based on these parameters, 73 of the 171 metabolites were recognized as KAIs. Moreover, although rutin (OB = 3.2%, DL ≥ 0.68) did not meet the screening criteria, it was classified as a KAI due to its extensively reported health-enhancing effects [33,34]. Finally, 74 metabolites in *D. officinale* were identified as KAIs associated with TCM (Supplementary Table S2). Among these, 34 were flavonoids, followed by 10 others, 9 terpenoids, 7 lignans and coumarins, 5 alkaloids, 5 phenolic acids, 2 quinones, 1 steroid, and 1 tannin. This indicated that flavonoids were the predominant bioactive group in *D. officinale*, followed by terpenoids, both contributing significantly to their pharmacological effects. Several metabolites displayed plant-part-specific distribution. Three were unique to the stems (acanthoside D, fargesin, and rhapontigenin), 1 to the leaves (gardenoside), and 12 to the flowers, including quercetin, geniposidic acid, kaempferol-3-*O*-galactoside, carthamone, homoeriodictyol 7-*O*-glucoside, coniferin, eriodictyol-7-*O*-glucoside, nepetin-7-*O*-glucoside, hemerocallin, polyporenic acid C, skimmin, and kaurenoic acid.

Among the 74 KAIs, 45 were associated with resistance to at least one disease. This finding significantly enriches the potential medicinal ingredient library of *D. officinale*. Seven metabolites, including quercetin, naringenin, robinetin, 2′,3′,4′,5,7-pentahydroxyflavone, acacetin, 3′-methoxydaidzein, and 2-methoxy-9,10-dihydrophenanthrene-4,5-diol, are linked to diseases with at least 90 entries. This indicated their high potential for health-related applications, warranting further investigation. The remaining 29 metabolites have not yet been linked to any target proteins or diseases. However, 11 of these metabolites exhibited remarkably high DL (≥0.72). Among these, six compounds—polyporenic acid C, hesperetin-5-O-glucoside, isoschaftoside, tricin-7-*O*-glucoside, apigenin 6,8-di-*C*-α-L-arabinopyranoside, and 3,3′-di-*O*-methylellagic acid 4′-glucoside—exhibited DL values > 0.8, indicating their potential as promising candidates for functional studies.

### 3.4. Identification of the Active Pharmaceutical Ingredients Relevant to Six Major Diseases in Dendrobium officinale

Cancer/tumor, diabetes, Alzheimer’s disease, hypertension, cardiovascular disease, and asthma are widely recognized as major threats to human health. All 761 identified metabolites were queried using the TCMSP database to identify APIs in *D. officinale* with potential activity against these 6 diseases. The results revealed that 83 metabolites were predicted by the TCMSP database to be APIs associated with resistance to at least one disease mentioned earlier (Supplementary Table S3). These 83 metabolites included 30 flavonoids, 17 others, 14 alkaloids, 8 phenolic acids, 7 terpenoids, 4 lignans and coumarins, 2 quinones, and 1 steroid. Among these, three metabolites (methyl caffeate, rhapontigenin, and *N*,*N*-dimethyl-5-methoxytryptamine) were exclusively detected in the stems and one (phloroglucinol) in the leaves. Additionally, 16 metabolites were detected exclusively in flowers, including flavonoids (quercetin, isorhamnetin-3-*O*-glucoside, chalcone, carthamone, kaempferol-3-*O*-glucoside, kaempferol-3-*O*-galactoside, scutellarein-7-*O*-glucoside, kaempferol-7-*O*-glucoside, homoeriodictyol 7-*O*-glucoside, and nepetin-7-*O*-glucoside), alkaloids (tryptamine and hordenine), terpenoids (kaurenoic acid and polyporenic acid C), and other bioactive compounds (skimmin and abscisic acid).

Of the 83 identified metabolites, 62 were associated with Alzheimer’s disease, 56 with hypertension, 47 with cardiovascular disease, 31 with asthma, 30 with cancer/tumors, and 23 with diabetes. A considerable number of metabolites were associated with multiple diseases: 37 with more than 3 diseases, 24 with more than 4 diseases, and 8 with more than 5 diseases. Three metabolites, including quercetin, 2′,3′,4′,5,7-pentahydroxyflavone, and acacetin, were associated with all six diseases. This suggested that these metabolites might be the principal APIs responsible for the medicinal potential of *D. officinale*.

### 3.5. Differential Metabolite Analysis Among Different Plant Parts of Dendrobium officinale

Differential metabolite analysis was performed to compare metabolite profiles across *D. officinale* plant parts. Metabolites meeting the criteria of VIP ≥ 1 and |Log2FC| ≥ 1 were defined as differentially accumulated metabolites (DAMs). As presented in Supplementary Table S4, 369 DAMs were identified between DOL and DOS, comprising 175 metabolites with increased abundance and 194 with decreased abundance (Figure S3A). The comparison between DOF and DOS revealed 510 DAMs, comprising 311 upregulated and 199 downregulated metabolites (Figure S3B). Between DOF and DOL, 502 DAMs were found, with 299 upregulated and 203 downregulated (Figure S3C). Among these, the metabolite displaying the highest fold-change increase in the DOL vs. DOS comparison was myricetin3-rhamnosyl-(1→3)-glucosyl-(1→6)-glucoside, whereas dehydrololiolide showed the most significant decrease in abundance (Figure S3D). Luteolin-7-*O*-(6″-malonyl) glucoside was most significantly upregulated, whereas homosyringic acid 4′-*O*-glucoside was most significantly downregulated in the DOF group compared with the DOS group (Figure S3E). Tamarixetin-3-*O*-(6″-malonyl) glucoside was the most significantly downregulated metabolite, whereas solatuberenol A was the most significantly downregulated metabolite in the DOF group compared with the DOL group (Figure S3F). Most of the upregulated metabolites in the leaves and flowers, compared with the stems, were flavonoid glycosides, such as myricetin and luteolin derivatives.

**Figure 3:**
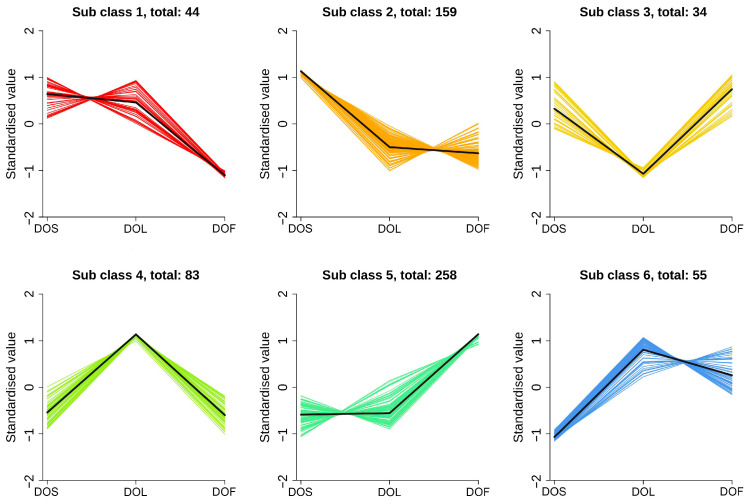
K-means clustering of metabolites based on relative accumulation patterns across stems (DOS), leaves (DOL), and flowers (DOF) of *D. officinale*. The horizontal axis represents the three plant parts, and the vertical axis shows the *Z*-score of relative metabolite abundance. Subclasses 1–6 were defined based on K-means clustering of metabolites with similar accumulation patterns. Prior to clustering, metabolite intensities were normalized and Z-score transformed. Statistical analyses were performed on three biological replicates (*n* = 3).

The heatmap analysis revealed distinct plant-part-specific metabolic profiles. DOL exhibited higher levels of flavonoids, whereas DOS were relatively enriched in phenolic acids, alkaloids, lignans, coumarins, terpenoids, quinones, and others (Figure S4A). The comparison between DOF and DOS revealed a greater abundance of flavonoids and alkaloids in DOF and more lignans and coumarins, quinones, and others in DOS (Figure S4B). DOF were enriched in flavonoids, alkaloids, terpenoids, and phenolic acids, whereas DOL exhibited higher levels of quinones and others (Figure S4C). Furthermore, K-means clustering was applied to categorize DAMs into six groups (Figure 3), comprising 44, 159, 34, 83, 258, and 55 DAMs in subclasses 1–6, respectively. Subclasses 1 and 2 were predominantly enriched in the stems, subclasses 4 and 6 in the leaves, and subclasses 3 and 5 in flowers. Subclass 5, the largest group (258 DAMs), included 25 KAIs and 30 APIs, notably quercetin and kaurenoic acid. Among the six groups, flavonoids and alkaloids were particularly abundant in leaves and flowers. They accounted for more than 50% of the total metabolites in subclasses 4 and 5, respectively, and nearly 80% in subclass 6.

**Figure 4:**
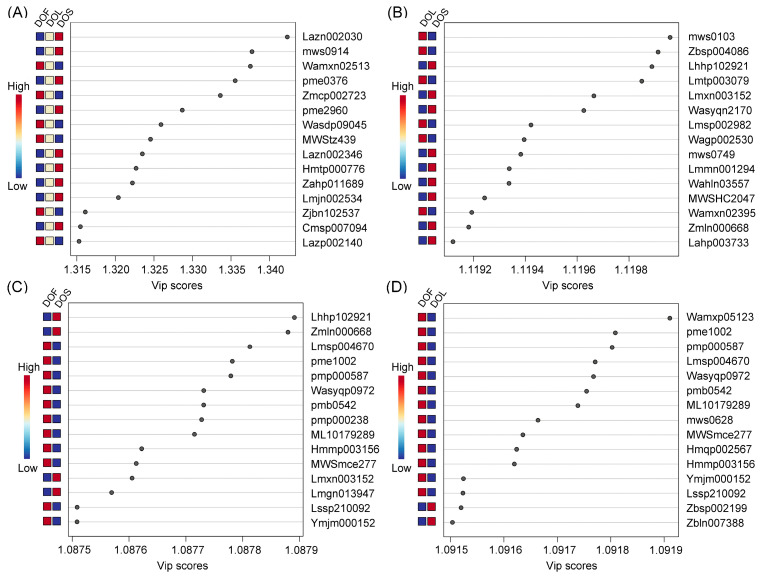
VIP score plots showing metabolic differences among stems (DOS), leaves (DOL), and flowers (DOF) in *D. officinale*. (**A**–**D**) Top 15 VIP-ranked metabolites in the overall comparison among DOF, DOL, and DOS (**A**) and in pairwise comparisons: DOL vs. DOS (**B**); DOF vs. DOS (**C**); and DOF vs. DOL (**D**). VIP scores were calculated from PLS-DA. Data represent three biological replicates (*n* = 3), and metabolite intensities were normalized and log-transformed prior to analysis. The color scale bar on the left indicates relative metabolite abundance, ranging from low (blue) to high (red). The identifiers shown on the *X*-axis (e.g., Lazn002030, mws0914) represent metabolite IDs assigned by the metabolomics platform. The full compound names corresponding to these IDs are provided in Supplementary Table S1.

**Figure 5:**
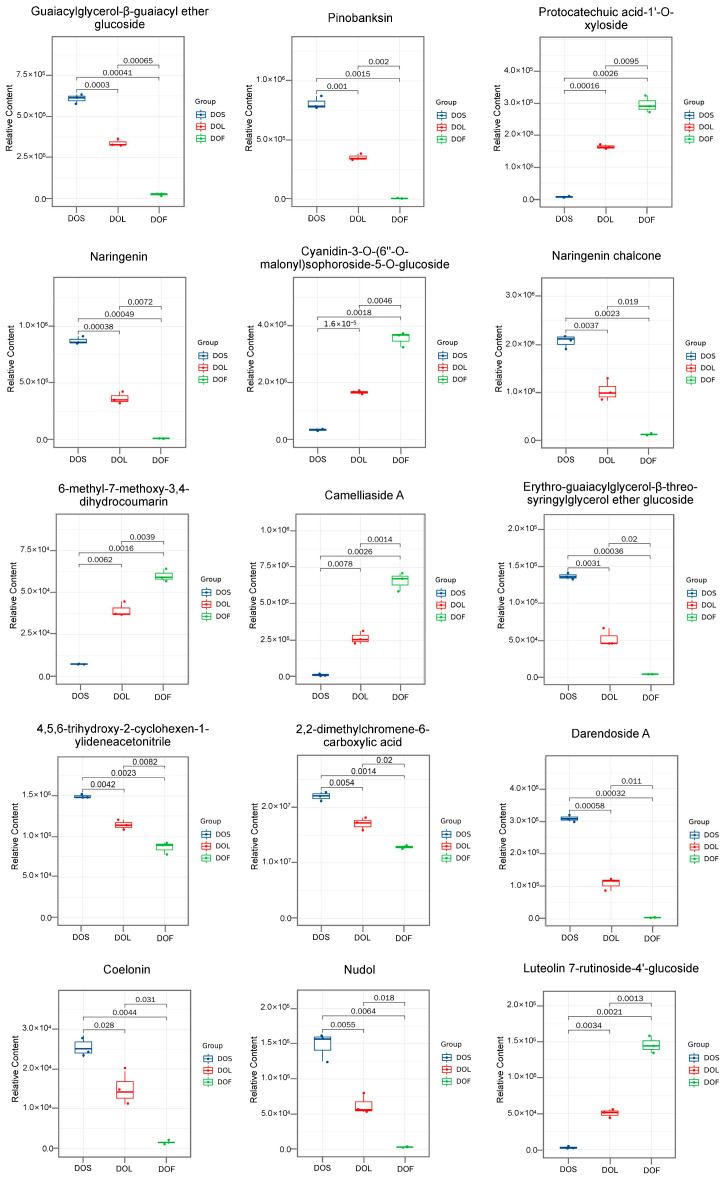
Distribution of the top 15 VIP-ranked metabolites among stems (DOS), leaves (DOL), and flowers (DOF) in *D. officinale*. Numbers above boxes indicate *p* values from pairwise Student’s *t* tests. Data are expressed as mean ± SD (*n* = 3). A *p* value less than 0.05 indicated a statistically significant difference.

Partial least squares discriminant analysis (PLS-DA) was performed for DOF vs. DOL vs. DOS, as well as for pairwise comparisons (DOL vs. DOS, DOF vs. DOS, and DOF vs. DOL), to further distinguish the metabolic differences among the three plant parts. The top 15 metabolites with VIP ≥ 1 were identified as key contributors to plant part discrimination. Overall comparison among DOF, DOL, and DOS revealed the three highest VIP-ranked metabolites: guaiacylglycerol-β-guaiacyl ether glucoside, pinobanksin, and protocatechuic acid-1′-*O*-xyloside (Figure 4A), highlighting the prominent role of flavonoids and phenolic glycosides in plant part differentiation. In the pairwise comparisons, the three highest-ranking metabolites for each comparison were: indole-3-carboxaldehyde, apigenin-6-*C*-glucose-8-*C*-rhamnoside, and magnolin for DOL vs. DOS (Figure 4B); magnolin, monogalloyl-diglucose, and kaempferol-3-*O*-glucoside-7-*O*-rhamnoside for DOF vs. DOS (Figure 4C); and (3R)-hydroxy-β-ionone malonylglucoside, L-tyramine, and luteolin-7-*O*-(6″-malonyl)glucoside for DOF vs. DOL (Figure 4D). The top 15 metabolites with VIP scores ≥ 1 from the overall comparison among DOF, DOL, and DOS were visualized using box plots to illustrate their distinct part-specific accumulation patterns (Figure 5). Naringenin, naringenin chalcone, pinobanksin, coelonin and nudol were predominantly accumulated in DOS, exhibiting levels 2.4, 2.0, 2.3, 1.7 and 2.4 times higher than those in DOL, respectively, and were nearly absent in DOF. In contrast, cyanidin-3-*O*-(6″-*O*-malonyl) sophoroside-5-*O*-glucoside, protocatechuic acid-1′-*O*-xyloside, camelliaside A and luteolin 7-rutinoside-4′-glucoside were highly enriched in DOF, with levels 2.1, 1.8, 2.5 and 2.9 times higher than those in DOL, respectively. However, these compounds were almost undetectable in DOS. These metabolites provide a chemical basis for plant-part-specific metabolic differentiation.

### 3.6. KEGG Analysis

**Figure 6:**
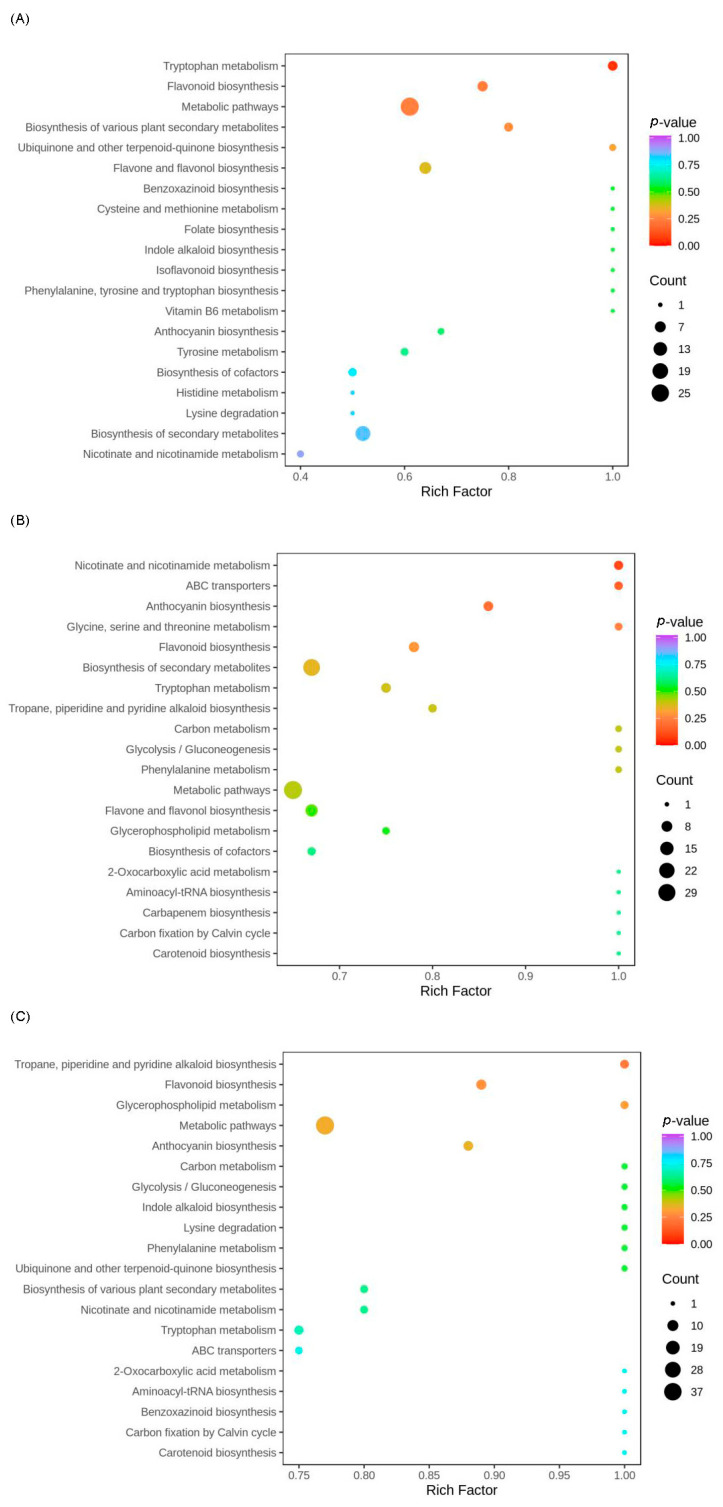
KEGG pathway enrichment analysis of differentially accumulated metabolites between plant part pairs: (**A**) leaves vs. stems; (**B**) flowers vs. stems; and (**C**) flowers vs. leaves. Each bubble represents a metabolic pathway. Bubble color indicates the significance level (*p* value), and bubble size reflects the number of enriched metabolites.

The KEGG metabolic pathway database, which provides graphical representations of numerous cellular biosynthesis and degradation pathways [35], was used to elucidate the potential biological functions of DAMs among *D. officinale* stems, leaves, and flowers. A total of 26, 51, and 53 metabolic pathways were significantly enriched in the DOL vs. DOS, DOF vs. DOS, and DOF vs. DOL comparisons, respectively. For each comparison, the top 20 enriched pathways are displayed in the respective KEGG bubble plots (Figure 6).

In the DOL vs. DOS comparison (Figure 6A), DAMs were predominantly enriched in the tryptophan metabolism (Ko00380) and flavonoid biosynthesis (Ko00941) pathways. Most metabolites in these two pathways exhibited higher accumulation levels in DOS than in DOL, including indole, 3-indoleacetonitrile, phloretin, and naringenin. In the DOF vs. DOS comparison (Figure 6B), the most significantly enriched pathways were nicotinate and nicotinamide metabolism (Ko00760), anthocyanin biosynthesis (Ko00942), and flavonoid biosynthesis. Metabolites in nicotinate and nicotinamide metabolism and anthocyanin biosynthesis were more abundant in DOF than in DOS, including nicotinate and nicotinamide derivatives as well as anthocyanin glycosides. For DOF vs. DOL comparison (Figure 6C), significant enrichment occurred in tropane, piperidine, and pyridine alkaloid biosynthesis (Ko00960), flavonoid biosynthesis, and anthocyanin biosynthesis pathways. Anthocyanin-related metabolites were particularly abundant in DOF. These KEGG enrichment results revealed distinct metabolic partitioning among *D. officinale* stems, leaves, and flowers.

### 3.7. Potential Biomarker Analysis in Dendrobium officinale Stems, Leaves, and Flowers

A comparative metabolomic analysis was performed to identify potential biomarkers among *D. officinale* stems, leaves, and flowers. Plant-part-specific biomarkers were defined as metabolites with at least a 2-fold higher or 0.5-fold lower abundance compared with other plant parts. This criterion was based on previously reported methods and standards [36].

**Figure 7:**
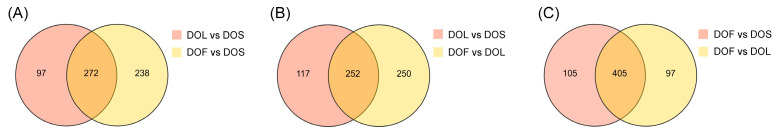
Venn diagrams of the differential metabolites. (**A**) Stem (DOS)-related groups. (**B**) Leaf (DOL)-related groups. (**C**) Flower (DOF)-related groups. Metabolites are selected based on VIP ≥ 1 and absolute log2FC ≥ 1 from three biological replicates (*n* = 3).

**Figure 8:**
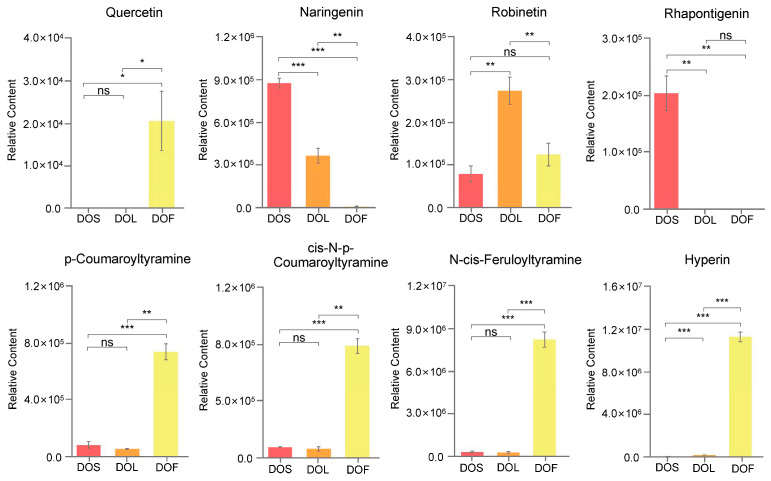
Relative content of eight key biomarkers among stems (DOS), leaves (DOL), and flowers (DOF) in *D. officinale*. Data are expressed as mean ± standard deviation (*n* = 3). Statistical significance between plant parts was assessed using Student’s *t* test (ns > 0.05, * *p* < 0.05, ** *p* < 0.01, *** *p* < 0.001).

DAMs were further analyzed to identify potential biomarkers in different plant parts of *D. officinale*. The Venn diagram analysis revealed that 272 metabolites were shared between DOS-related comparisons (DOL vs. DOS, DOF vs. DOS), 252 metabolites were common to all DOL-related comparisons (DOL vs. DOS, DOF vs. DOL), and 406 metabolites were shared between DOF-related comparisons (DOF vs. DOS, DOF vs. DOL) (Figure 7A–C). All metabolites exhibited significant differences (|log_2_FC| ≥ 1). Among these, 125, 73, and 243 metabolites were preferentially accumulated in DOS, DOL, and DOF, respectively. These DAMs were considered potential biomarkers, several of which had been previously identified as KAIs and APIs. Potential biomarkers are summarized in Supplementary Table S5. Eight metabolites were identified as both KAIs and APIs, each associated with more than 50 disease-related entries. These included quercetin, naringenin, robinetin, rhapontigenin, p-coumaroyltyramine, *cis*-N-p-coumaroyltyramine, N-*cis*-feruloyltyramine, and hyperin. They are considered the most prominent and functionally significant metabolites across the plant parts of *D. officinale*. The relative content of these eight key biomarkers in DOS, DOL, and DOF is presented in Figure 8, and their retention times and representative MS/MS fragment ions are provided in Supplementary Table S6. Quercetin, p-coumaroyltyramine, *cis*-N-p-coumaroyltyramine, N-*cis*-feruloyltyramine, and hyperin were highly enriched in DOF, whereas their levels in DOS and DOL were negligible. In contrast, rhapontigenin accumulated predominantly in DOS, with negligible levels in DOL and DOF. Naringenin was most abundant in DOS, with a concentration approximately twice that of DOL, and was nearly undetectable in DOF. In contrast, robinetin was enriched in DOL, reaching levels 3.5 times higher than those in DOS and 2.2 times higher than those in DOF, respectively. The accumulation of these metabolites was highly part-specific.

## 4. Discussion

*Dendrobium officinale* Kimura et Migo has been widely cherished in traditional Chinese medicine (TCM) for its pronounced therapeutic efficacy and diverse pharmacological action. To the best of our knowledge, this study presents the first systematically characterized secondary metabolite profiles in the stems, leaves, and flowers of *D. officinale*. The results revealed pronounced plant-part-specific differences in metabolite composition and abundance, thereby highlighting the distinct biochemical and pharmacological characteristics of each plant part. Among all plant parts, the flowers contained the highest abundance of metabolites, followed by leaves and stems, suggesting that metabolite diversity correlates with part differentiation and physiological function, providing essential information for the rational use of all plant parts. Within the metabolites identified in this study, flavonoids and alkaloids were the most abundant classes, together accounting for more than half of all detected compounds. This distribution was consistent with previous findings [12,37,38], confirming the central roles of these two classes in the pharmacological efficacy of *D. officinale*.

The highest accumulation of flavonoids and alkaloids was observed in flowers, consistent with the findings of Tang et al. [3]. This enrichment might be attributed to the specific physiological roles of flowers, which often require enhanced protection against ultraviolet radiation and oxidative stress, as well as the synthesis of pigments and scent compounds to attract pollinators [39]. The increased expression of key biosynthetic genes, such as CHS, F3H, and FLS, has been reported in floral parts of many plant species, contributing to the greater accumulation of flavonoids and related secondary metabolites [40]. Similarly, flowers often function as primary sites for the biosynthesis or storage of specific alkaloids, contributing to plant defense and reproductive success. This may explain their higher content compared with that in other plant parts [41]. This specialized biosynthetic activity explains the higher content of alkaloids and flavonoids in flowers compared with stems and leaves, reflecting differences between plant parts.

Screening against the TCMSP database identified 74 KAIs with potential health-promoting effects. Flavonoids constituted the most abundant class, followed by terpenoids, indicating their major role in pharmacological activity. Among flavonoids, naringenin, quercetin, rutin, isovitexin, and hyperin are well documented for their antioxidant, anti-inflammatory, and neuroprotective properties [42,43,44,45]. Terpenoids such as ferruginol, kaurenoic acid, geniposidic acid, and geniposide exhibit multiple bioactivities, including anti-inflammatory, antioxidant, and anticancer effects [46,47,48].

The differences between plant parts were clearly evident for these metabolites. Gardenoside was detected exclusively in leaves, whereas quercetin and geniposidic acid were particularly abundant in flowers, emphasizing the expansion of the medicinal library beyond the stem. Quercetin and geniposidic acid were particularly abundant in flowers and notable for their strong pharmacological activities. Quercetin is widely recognized as one of the most potent natural antioxidants and anti-inflammatory agents in plants. It effectively scavenges free radicals, modulates inflammatory pathways, and exerts neuroprotective and cardiovascular protective effects [46,49,50]. Geniposidic acid also exhibits multiple biological activities, including hepatoprotective, anti-inflammatory, and neuroprotective effects. These activities are largely attributed to its ability to suppress oxidative stress and regulate inflammatory cytokine expression [51,52]. Collectively, these KAIs illustrate the pharmacologically relevant diversity across plant parts and underscore the value of non-stem plant parts for drug discovery. Leaves and flowers contain comparable or greater numbers of metabolites than stems, including unique bioactive compounds, challenging the traditional stem-focused view of *D. officinale*.

Further in silico analysis identified 83 APIs relevant to 6 major diseases. This TCMSP-based screening directly maps the metabolome of *D. officinale* to potential therapeutic actions against Alzheimer’s disease, hypertension, cardiovascular disease, asthma, cancer/tumors, and diabetes, and identifies core multi-disease agents such as quercetin, 2′,3′,4′,5,7-pentahydroxyflavone, and acacetin. For example, phloroglucinol, abundant in leaves, is predicted to exert antioxidant, anti-inflammatory, and antimicrobial effects by regulating the AMPK/Nrf2/HO-1 signaling pathway [53,54]. In flowers, chalcone and kaempferol glycosides were enriched with antioxidant, anti-inflammatory, and cardiovascular and neuroprotective effects [55,56,57]. These findings reinforce that leaves and flowers contain valuable medicinal components, complementing the traditional use of stems, thereby broadening the functional applications of *D. officinale*.

Interestingly, multivariate statistical analyses further support these differences between plant parts. Both PCA and PLS-DA models revealed clear separation among the plan parts. Metabolites such as naringenin and naringenin chalcone were most abundant in stems, consistent with their roles in flavonoid metabolism and structural defense [58]. However, cyanidin-3-*O*-(6″-*O*-malonyl)sophoroside-5-*O*-glucoside was enriched in flowers, potentially contributing to pigmentation and enhanced oxidative stress resistance [59]. KEGG enrichment analysis revealed that pathways such as flavonoid biosynthesis, tryptophan metabolism, nicotinate and nicotinamide metabolism, and anthocyanin biosynthesis were predominantly involved in the metabolic differences among the different plant parts. Higher accumulation of indole and related derivatives in stems suggested enhanced tryptophan metabolism, supporting auxin biosynthesis, growth regulation, and stress tolerance [60,61]. Elevated flavonoid and anthocyanin contents in flowers likely contribute to pigmentation, reproductive success, and antioxidant capacity, whereas increased NAD^+^-related metabolite levels might be associated with stress-response pathways [62,63], although this was not directly measured in the present study. Furthermore, the study provided functional and pharmacological insights into plant-part-specific biomarkers. Among these metabolites, quercetin, p-coumaroyltyramine, *cis*-N-p-coumaroyltyramine, N-*cis*-feruloyltyramine, and hyperin were enriched in flowers, naringenin and rhapontigenin in stems, and robinetin in leaves. These findings reinforce the concept of metabolic specialization among plant parts and underscore the underexplored potential of leaves and flowers for medicinal and nutritional applications. Future studies incorporating absolute quantification of key metabolites, along with new in vitro and in vivo assays, are warranted to further refine our understanding of their functional relevance.

Moreover, the detailed metabolomic profiling allows us to characterize the chemical composition of *D. officinale* across its major plant parts, including stems, leaves, and flowers. By identifying metabolites enriched in each plant part, the analysis reveals which parts contain notable bioactive compounds, thereby facilitating the comprehensive utilization of the entire plant and guiding targeted harvesting and processing strategies. Such knowledge enables more efficient use of the entire plant and guides targeted harvesting and processing strategies to maximize pharmacological and nutritional benefits.

Overall, this integrative metabolomic study provides a comprehensive framework for understanding the chemical diversity of *D. officinale*. Our results highlight that flowers and leaves are rich sources of bioactive metabolites, revealing plant-part-specific chemical patterns and therapeutic potentials in these non-stem parts. This research not only advances our understanding of *D. officinale*’s chemical diversity but also provides a chemical foundation for future precision-based extraction strategies and informs the potential for broader utilization of this medicinal plant. These insights also inform the potential broader utilization of the plant, enhancing resource efficiency and guiding subsequent functional studies and targeted product development.

## 5. Conclusions

In this study, a comprehensive metabolomic analysis of *D. officinale* stems, leaves, and flowers was conducted using UHPLC–MS/MS integrated with network pharmacology. A total of 761 metabolites were identified, predominantly consisting of flavonoids (30.6%), alkaloids (20.2%), phenolic acids (12.2%), and terpenoids (9.3%). Notably, leaves and flowers accumulated higher levels of flavonoids and alkaloids than stems, underscoring their potential as enriched sources of bioactive compounds. Among the identified metabolites, 74 were KAIs and 83 APIs were detected, with flowers showing the highest enrichment. Specifically, 12 KAIs and 16 APIs were unique to flowers, while gardenoside and phloroglucinol were exclusively found in leaves. Potential biomarkers, including quercetin, naringenin, robinetin, hyperin, and rhapontigenin, were also identified. Quercetin and hyperin, detected exclusively in flowers, have been recognized for their anti-inflammatory and cardioprotective properties, reinforcing the therapeutic value of this species. The plant-part-specific distribution of metabolites observed in this study provides a scientific foundation for the pharmaceutical and nutritional exploitation of *D. officinale*. Given that leaves and flowers remain underutilized in current applications, our findings suggest that these plant parts could serve as novel sources of high-value bioactive compounds for drug development and functional food applications. This research not only advances our understanding of *D. officinale*’s chemical diversity but also opens new avenues for precision-based extraction and utilization strategies, thereby enhancing the economic and therapeutic potential of this medicinal plant.

## Data Availability

Data associated with this research can be accessed by contacting the corresponding author with a reasonable request.

## Supplementary Materials

The following supporting information can be downloaded at https://www.mdpi.com/article/10.3390/metabo16010010/s1. Figure S1: QC and sample chromatograms of *D. officinale* in positive and negative ion modes. (**A**,**B**) QC-TIC diagram. (**C**,**D**) Sample multi-peak detection diagram; Figure S2: Comparative metabolite analysis in *D. officinale*. (**A**) Venn diagram across different plant parts. (**B**) Hierarchical clustering heatmap of metabolite profiles. The color gradient reflects the relative levels of each metabolite, with green indicating low abundance and red indicating high abundance. Sample labels DOS (1–3), DOL (1–3), and DOF (1–3) represent three biological replicates (*n* = 3) of stems (DOS), leaves (DOL), and flowers (DOF), respectively; Figure S3: Metabolite differences between plant parts in *D*. *officinale* (DOS: stem, DOL: leaf, DOF: flower). (**A**–**C**) Volcano plots for DOL vs. DOS, DOF vs. DOS, and DOF vs. DOL, respectively. Red, green, and gray dots denote significantly upregulated, downregulated, and nonsignificant metabolites, respectively. (**D**–**F**) Top five upregulated and downregulated metabolites for DOL vs. DOS, DOF vs. DOS, and DOF vs. DOL, respectively. Analyses were performed on three biological replicates (*n* = 3); Figure S4: Hierarchical clustering heatmap of differentially accumulated metabolites between plant parts in *D*. *officinale* (DOS: stem, DOL: leaf, DOF: flower): (**A**) DOL vs. DOS, (**B**) DOF vs. DOS, and (**C**) DOF vs. DOL. Each column corresponds to a sample, and each row represents a metabolite. High levels are shown in red, and low levels in green. Analyses were performed on three biological replicates (*n* = 3); Supplementary Table S1: Metabolites identified and quantified in *D. officinale*; Supplementary Table S2: Key active ingredients identified in *D. officinale*; Supplementary Table S3: Active pharmaceutical ingredients identified in *D. officinale*; Supplementary Table S4: Differentially accumulated metabolites among stems (DOS), leaves (DOL), and flowers (DOF) in *D. officinale*; Supplementary Table S5: Potential biomarkers identified in stems (DOS), leaves (DOL), and flowers (DOF) of *D. officinale*; Supplementary Table S6: Retention times and MS/MS fragments of eight key biomarkers among stems, leaves, and flowers in *D. officinale*.

## Abbreviations

The following abbreviations are used in this manuscript:

APIs	Active pharmaceutical ingredients
DAMs	Differentially accumulated metabolites
DL	Drug-likeness
DOF	Flowers of *Dendrobium officinale*
DOL	Leaves of *Dendrobium officinale*
DOS	Stems of *Dendrobium officinale*
HCA	Hierarchical clustering analysis
KAIs	Key active ingredients
MRM	Multiple reaction monitoring
OB	Oral bioavailability
OPLS-DA	Orthogonal partial least squares discriminant analysis
PCA	Principal component analysis
PCC	Pearson correlation analysis
PLS-DA	Partial least squares discriminant analysis
QC	Quality control
QTRAP	Quadrupole ion trap tandem mass spectrometry
TCM	Traditional Chinese medicine
TCMSP	Traditional Chinese Medicine Systems Pharmacology Database and Analysis Platform
TIC	Total ion chromatogram
UHPLC–MS/MS	Ultra-high-performance liquid chromatography–tandem mass spectrometry
API	Active pharmaceutical ingredients
DAM	Differentially accumulated metabolites
