# Supplementary material for: Bioactive Ingredient Profiling of Dendrobium officinale: Plant-Part-Specific Distribution of Key Metabolites and Their Multi-Disease Therapeutic Potential
Source: Metabolites. 2025 Dec 22;16(1):10. doi: 10.3390/metabo16010010 (PMC12844509; doi:10.3390/metabo16010010)
Supplement: Supplementary file 1 [file metabolites-16-00010-s001.zip › Revised Supplementary data-1.pdf]

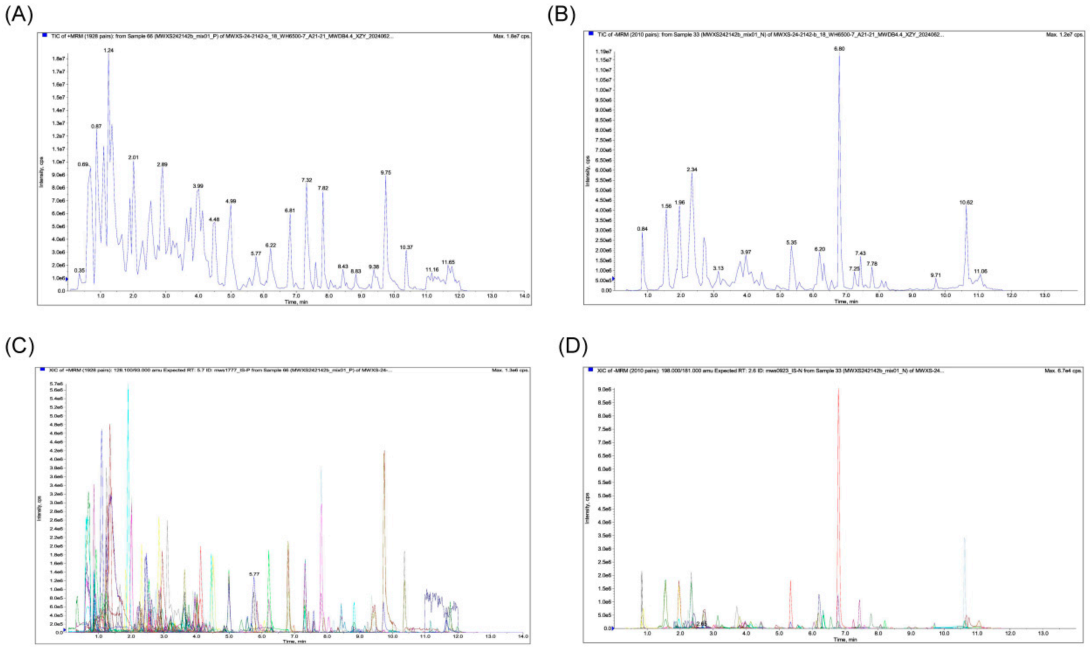

Figure S1. QC and sample chromatograms of *D. officinale* in positive and negative ion modes. (**A** and **B**) QC-TIC diagram. (**C** and **D**) Sample multi-peak detection diagram.

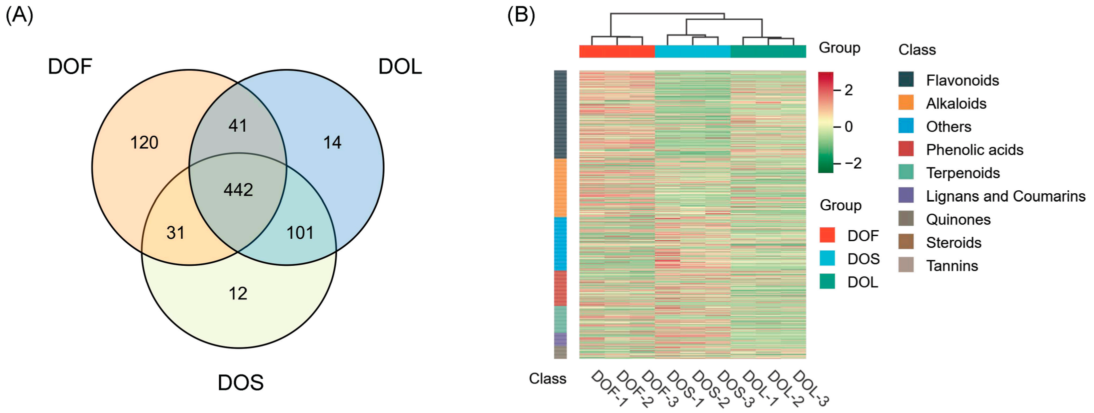

Figure S2. Comparative metabolite analysis in *D. officinal*e. (**A**) Venn diagram across diffent plant parts. (**B**) Hierarchical clustering heatmap of metabolite profiles. The color gradient reflects the relative levels of each metabolite, with green indicating low abundance and red indicating high abundance. Sample labels DOS (1–3), DOL (1–3), and DOF (1–3) represent three biological replicates (*n*=3) of stems (DOS), leaves (DOL), and flowers (DOF), respectively.

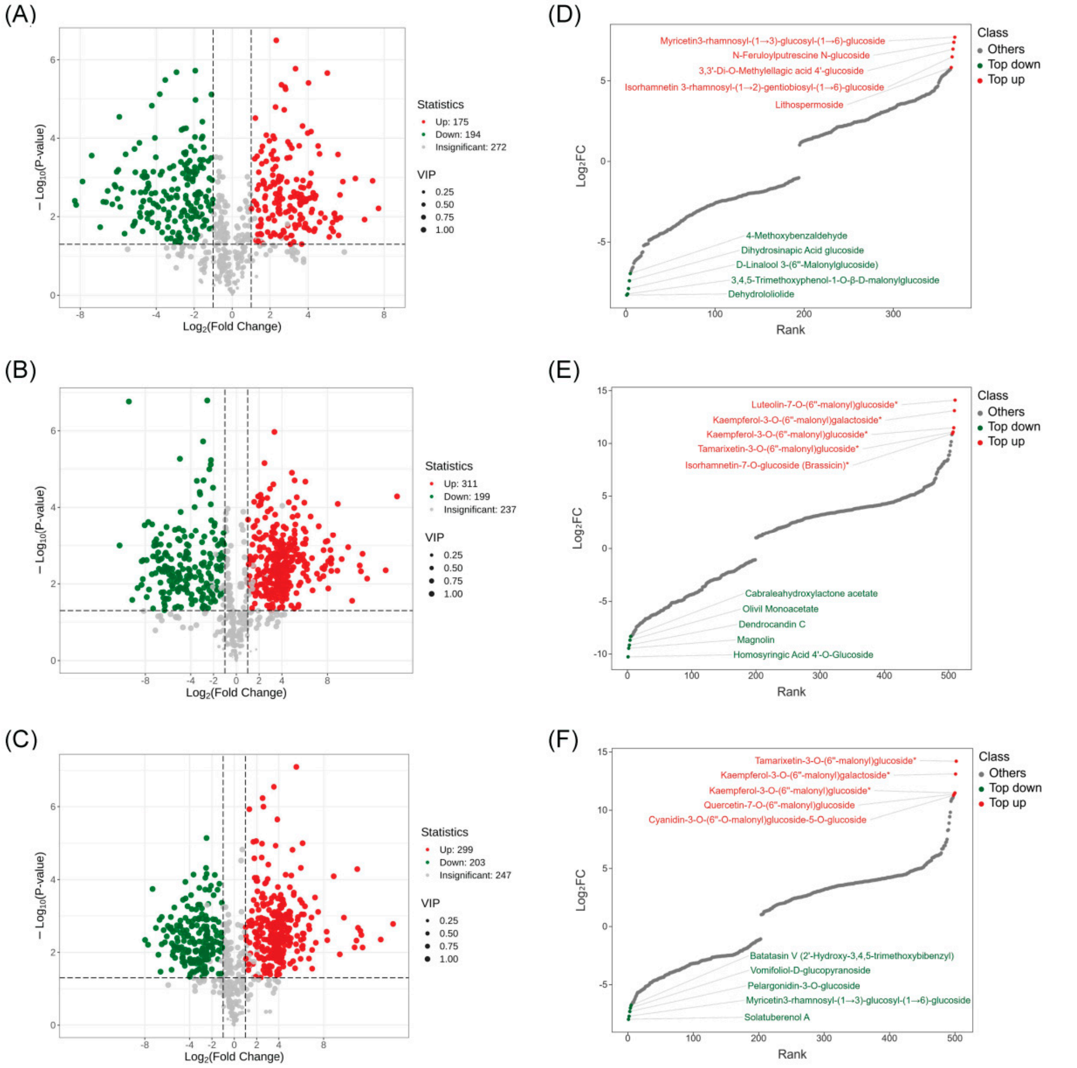

Figure S3. Metabolite differences between plant parts in *D. officinale* (DOS: stem, DOL: leaf, DOF: flower). (**A–C**) Volcano plots for DOL vs. DOS, DOF vs. DOS, and DOF vs. DOL, respectively. Red, green, and gray dots denote significantly upregulated, downregulated, and nonsignificant metabolites, respectively. (**D–F**) Top five upregulated and downregulated metabolites for DOL vs. DOS, DOF vs. DOS, and DOF vs. DOL, respectively. Analyses were performed on three biological replicates ($n = 3$).

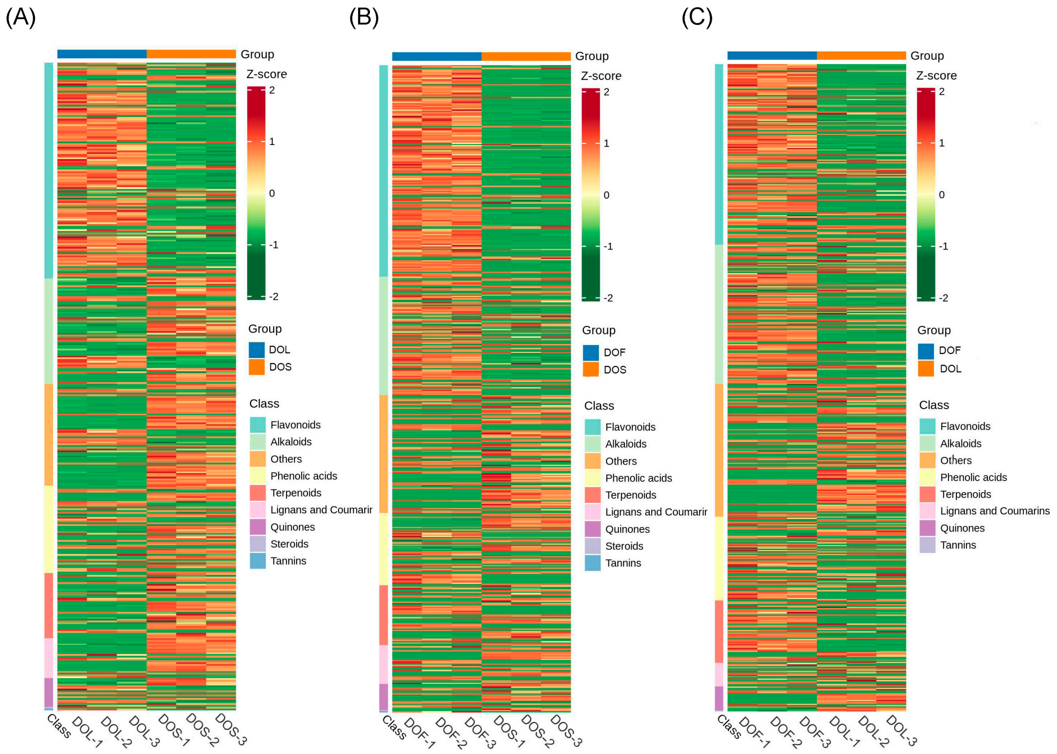

Figure S4. Hierarchical clustering heatmap of differentially accumulated metabolites between plant parts in *D. officinale* (DOS: stem, DOL: leaf, DOF: flower): (**A**) DOL vs. DOS, (**B**) DOF vs. DOS, and (**C**) DOF vs. DOL. Each column corresponds to a sample, and each row represents a metabolite. High levels are shown in red, and low levels in green. Analyses were performed on three biological replicates ($n = 3$).
